# Supplementary material for: From sequence to enzyme mechanism using multi-label machine learning
Source: BMC Bioinformatics. 2014 May 19;15:150. doi: 10.1186/1471-2105-15-150 (PMC4229970; doi:10.1186/1471-2105-15-150)
Supplement: Additional file 2 — Java code of ml2db. Additional file ml2db_code.tar.gz contains the Java source code to run the multi-label machine learning experiments and save the results to database. The code’s Javadoc is included. [file 1471-2105-15-150-S2.zip › additional file 2/ml2db/utils/doc/uk/ac/standrews/utils/main/database/DbConn.html]

DbConn


JavaScript is disabled on your browser.


- Overview
- Package
- Class
- Use
- Tree
- Deprecated
- Index
- Help

- Prev Class
- Next Class

- Frames
- No Frames

- All Classes

- Summary:
- Nested |
- Field |
- Constr |
- Method

- Detail:
- Field |
- Constr |
- Method


uk.ac.standrews.utils.main.database

## Class DbConn

- java.lang.Object
- - uk.ac.standrews.utils.main.database.DbConn

- All Implemented Interfaces:
  :   java.io.Serializable

  ---

    

  ```
  public class DbConn
  extends java.lang.Object
  implements java.io.Serializable
  ```

  A DbConnectionProperties object contains the properties needed to connect to
  a database through JDBC. The properties are read from the default properties
  file or from a given file.
  DbConnectionProperties also generates a new connection and statement.
  The Java properties file is a text file in the form:
  # Database type databaseType=mysql # JDBC driver (comma-separated list)
  jdbcDriver=org.gjt.mm.mysql.Driver # Connection parameters host=localhost
  port=3306 database=mydb user=myuser pswd=secretpassword

  Version:
  :   18-Apr-2007

  Author:
  :   ldeferra

  See Also:
  :   Serialized Form

- - ### Field Summary

    Fields

    | Modifier and Type | Field and Description |
    | --- | --- |
    | `static java.lang.String` | `DATABASE_NAME_PROP` |
    | `static java.lang.String` | `DATABASE_TYPE_PROP` |
    | `static java.lang.String` | `HOST_PROP` |
    | `static java.lang.String` | `JDBC_DRIVER_PROP` |
    | `static java.lang.String` | `PASSWORD_PROP` |
    | `static java.lang.String` | `PORT_PROP` |
    | `static java.lang.String` | `USER_PROP` |
  - ### Constructor Summary

    Constructors

    Constructor and Description

    `DbConn(java.lang.String propertiesFilePath)`

    Constructor: takes connection parameters from the specified properties
    file
  - ### Method Summary

    Methods

    | Modifier and Type | Method and Description |
    | --- | --- |
    | `void` | `closeConnection()` Close the database connection |
    | `boolean` | `connectionIsValid()` |
    | `static boolean` | `connectionIsValid(java.sql.Connection connection)` Checks that the connection is not null |
    | `java.sql.Connection` | `getConnection()` |
    | `java.lang.String` | `getConnectionParametersString()` Get the connection log (database driver + url) |
    | `java.lang.String` | `getConnectionPropertiesFilePath()` |
    | `java.lang.String` | `getDatabaseName()` Gets the database the connection is connected to |
    | `java.lang.String` | `getDatabaseType()` |
    | `java.lang.String` | `getDriverName()` |
    | `java.lang.String` | `getHost()` |
    | `java.lang.String` | `getPassword()` |
    | `java.lang.String` | `getPort()` |
    | `java.sql.Statement` | `getStatement()` Gets a statement from a new connection |
    | `java.lang.String` | `getUrl()` |
    | `java.lang.String` | `getUser()` |
    | `void` | `readConnectionProperties(java.lang.String propertiesFilePath)` Reads the connection properties from the java properties file specified in the path |
    | `java.sql.Connection` | `regenerateConnection()` |

    - ### Methods inherited from class java.lang.Object

      `equals, getClass, hashCode, notify, notifyAll, toString, wait, wait, wait`

- - ### Field Detail


    - #### DATABASE\_NAME\_PROP

      ```
      public static final java.lang.String DATABASE_NAME_PROP
      ```

      See Also:
      :   Constant Field Values


    - #### DATABASE\_TYPE\_PROP

      ```
      public static final java.lang.String DATABASE_TYPE_PROP
      ```

      See Also:
      :   Constant Field Values


    - #### HOST\_PROP

      ```
      public static final java.lang.String HOST_PROP
      ```

      See Also:
      :   Constant Field Values


    - #### JDBC\_DRIVER\_PROP

      ```
      public static final java.lang.String JDBC_DRIVER_PROP
      ```

      See Also:
      :   Constant Field Values


    - #### PASSWORD\_PROP

      ```
      public static final java.lang.String PASSWORD_PROP
      ```

      See Also:
      :   Constant Field Values


    - #### PORT\_PROP

      ```
      public static final java.lang.String PORT_PROP
      ```

      See Also:
      :   Constant Field Values


    - #### USER\_PROP

      ```
      public static final java.lang.String USER_PROP
      ```

      See Also:
      :   Constant Field Values
  - ### Constructor Detail


    - #### DbConn

      ```
      public DbConn(java.lang.String propertiesFilePath)
      ```

      Constructor: takes connection parameters from the specified properties
      file
  - ### Method Detail


    - #### connectionIsValid

      ```
      public static boolean connectionIsValid(java.sql.Connection connection)
      ```

      Checks that the connection is not null

      Parameters:
      :   `connection` -

      Returns:
      :   true if the connection is not null or has not been closed


    - #### closeConnection

      ```
      public void closeConnection()
      ```

      Close the database connection


    - #### connectionIsValid

      ```
      public boolean connectionIsValid()
      ```


    - #### getConnection

      ```
      public java.sql.Connection getConnection()
      ```


    - #### getConnectionParametersString

      ```
      public java.lang.String getConnectionParametersString()
      ```

      Get the connection log (database driver + url)

      Returns:
      :   the connection log (database driver + url)


    - #### getConnectionPropertiesFilePath

      ```
      public java.lang.String getConnectionPropertiesFilePath()
      ```


    - #### getDatabaseName

      ```
      public java.lang.String getDatabaseName()
      ```

      Gets the database the connection is connected to

      Returns:
      :   the database name


    - #### getDatabaseType

      ```
      public java.lang.String getDatabaseType()
      ```


    - #### getDriverName

      ```
      public java.lang.String getDriverName()
      ```


    - #### getHost

      ```
      public java.lang.String getHost()
      ```


    - #### getPassword

      ```
      public java.lang.String getPassword()
      ```


    - #### getPort

      ```
      public java.lang.String getPort()
      ```


    - #### getStatement

      ```
      public java.sql.Statement getStatement()
      ```

      Gets a statement from a new connection

      Returns:
      :   a database statement


    - #### getUrl

      ```
      public java.lang.String getUrl()
      ```


    - #### getUser

      ```
      public java.lang.String getUser()
      ```


    - #### readConnectionProperties

      ```
      public void readConnectionProperties(java.lang.String propertiesFilePath)
      ```

      Reads the connection properties from the java properties file specified
      in the path

      Parameters:
      :   `propertiesFilePath` - the path to the java properties file containing the connection
          parameters


    - #### regenerateConnection

      ```
      public java.sql.Connection regenerateConnection()
      ```


- Overview
- Package
- Class
- Use
- Tree
- Deprecated
- Index
- Help

- Prev Class
- Next Class

- Frames
- No Frames

- All Classes

- Summary:
- Nested |
- Field |
- Constr |
- Method

- Detail:
- Field |
- Constr |
- Method
